# Associations between ambient air pollutant mixtures and pediatric asthma emergency department visits in three cities: a classification and regression tree approach

**DOI:** 10.1186/s12940-015-0044-5

**Published:** 2015-06-27

**Authors:** Katherine Gass, Mitch Klein, Stefanie E. Sarnat, Andrea Winquist, Lyndsey A. Darrow, W. Dana Flanders, Howard H. Chang, James A. Mulholland, Paige E. Tolbert, Matthew J. Strickland

**Affiliations:** Department of Epidemiology, Rollins School of Public Health, Emory University, 1518 Clifton Rd, Atlanta, GA 30322 USA; Department of Environmental Health, Rollins School of Public Health, Emory University, 1518 Clifton Rd, Atlanta, GA 30322 USA; Department of Biostatistics and Bioinformatics, Rollins School of Public Health, Emory University, 1518 Clifton Rd, Atlanta, GA 30322 USA; School of Civil and Environmental Engineering, Georgia Institute of Technology, 790 Atlantic Drive, Atlanta, Georgia 30332 USA

**Keywords:** Air pollution, Classification and regression trees, Multicity, Multipollutant, Mixtures, NO_2_, Ozone, Pediatric asthma, PM_2.5_

## Abstract

**Background:**

Characterizing multipollutant health effects is challenging. We use classification and regression trees to identify multipollutant joint effects associated with pediatric asthma exacerbations and compare these results with those from a multipollutant regression model with continuous joint effects.

**Methods:**

We investigate the joint effects of ozone, NO_2_ and PM_2.5_ on emergency department visits for pediatric asthma in Atlanta (1999–2009), Dallas (2006–2009) and St. Louis (2001–2007). Daily concentrations of each pollutant were categorized into four levels, resulting in 64 different combinations or “Day-Types” that can occur. Days when all pollutants were in the lowest level were withheld as the reference group. Separate regression trees were grown for each city, with partitioning based on Day-Type in a model with control for confounding. Day-Types that appeared together in the same terminal node in all three trees were considered to be mixtures of potential interest and were included as indicator variables in a three-city Poisson generalized linear model with confounding control and rate ratios calculated relative to the reference group. For comparison, we estimated analogous joint effects from a multipollutant Poisson model that included terms for each pollutant, with concentrations modeled continuously.

**Results and discussion:**

No single mixture emerged as the most harmful. Instead, the rate ratios for the mixtures suggest that all three pollutants drive the health association, and that the rate plateaus in the mixtures with the highest concentrations. In contrast, the results from the comparison model are dominated by an association with ozone and suggest that the rate increases with concentration.

**Conclusion:**

The use of classification and regression trees to identify joint effects may lead to different conclusions than multipollutant models with continuous joint effects and may serve as a complementary approach for understanding health effects of multipollutant mixtures.

## Introduction

Humans breathe a mixture of different air pollutants. Characterization of these multipollutant mixtures in relation to health effects has been addressed by air pollution research groups for decades. Historically, a common epidemiological approach for addressing mixtures has been through single pollutant models, in which a single pollutant effect is viewed as a surrogate for a particular air pollution mixture. Other approaches for investigating pollutant mixtures include composite metrics such as air quality indices [1], hierarchical and k-means clustering [2, 3], supervised principal components analysis [4], groupings based on chemical properties of pollutants [5], source apportionment [6], and exposure to specific mixtures, such as traffic-related pollutants [7].

Two reviews of statistical approaches for multipollutant research were recently published [8, 9]. In both reviews, classification and regression trees (C&RT), a supervised recursive partitioning approach, was cited as a method for handling multipollutant exposures; however, there have been few applications of C&RT in assessing the health effects of ambient air pollution exposure [10, 11]. In a recent paper we showed how C&RT can be adapted for epidemiologic research, particularly to control for confounding, and become a useful tool for generating hypotheses about multipollutant joint effects [12].

C&RT groups days according to their multipollutant profiles. This can help to elucidate patterns of meteorology, seasonality, and emission sources that cause certain pollutants to covary more strongly than others. From a health perspective, grouping days can enable identification of day types that are more harmful to human health and help to improve risk prediction systems. From a regulatory perspective, identifying the most harmful multipollutant joint effects can lead to more targeted regulation.

In this analysis, we use C&RT to identify multipollutant mixtures associated with pediatric asthma emergency department (ED) visits and calculate the joint effects associated with each mixture in a time series framework. Our analysis focuses on three criteria pollutants, ozone (O_3_), nitrogen dioxide (NO_2_), and particulate matter less than 2.5 microns in diameter (PM_2.5_), shown to have strong associations with asthma/wheeze in previous analyses [13, 14]. We conduct three separate C&RT analyses using time series data from Atlanta, Dallas and St. Louis. To increase statistical power we combined C&RT results across three cities. Although methods exist for combining trees generated from random subsets of a single data source (e.g. bagging and random forests), similarly well-established methods do not exist for combining trees generated from different data sources. In this paper we present an approach for combining C&RT results across cities to identify between-city similarities. A drawback of combining results from different cities is that city-specific heterogeneity will be masked. This concern is not unique to our study but rather is a generic epidemiologic concern that should be considered whenever estimates are pooled across strata, and in air pollution epidemiology there is a precedent of combining data across different cities, starting with the National Morbidity, Mortality and Air Pollution Study [15].

Finally, because C&RT is seldom used in multipollutant research, we compare the results of this three-city C&RT approach with the analogous joint effects estimated from a multipollutant regression model with each pollutant concentration modeled continuously [16].

## Methods

### Data

#### Emergency Department visit data

Computerized billing records for ED visits to acute care hospitals in each city were obtained as follows: for 20-county Atlanta, from individual hospitals and the Georgia Hospital Association for an 11-year study period (1/1/1999 – 12/31/2009); for 12-county Dallas, from the Dallas-Fort Worth Hospital Council Foundation for a 3.5-year study period (1/1/2006 – 8/31/2009); and for 16-county St. Louis, from the Missouri Hospital Association for a 6.5-year study period (1/1/2001 – 6/27/2007). Relevant data elements included a patient identifier, admission date, patient age, primary and secondary International Classification of Diseases 9^th^ Revision (ICD-9) diagnosis codes, and ZIP code of patient residence. Data were used in accordance with the individual hospital and/or hospital association data use agreements; this study was also approved by the Emory University Institutional Review Board. Visits by patients living in ZIP codes outside the city-specific study areas were excluded.

The individual-level data were restricted to visits by pediatric patients ages 2–18 years and aggregated to daily counts of asthma and/or wheeze, identified as any ICD-9 code of 493 and/or 786.07, resulting in 271,725 visits in Atlanta, 116,212 in Dallas, and 100,471 in St. Louis.

#### Air pollution data

Daily population-weighted average concentrations of ambient O_3_ (8-hr max), NO_2_ (1-hr max) and PM_2.5_ (24-h average) were calculated using measurements from stationary monitors in each of the three cities [17]. The averaging times for each pollutant were selected to coincide with those used for the National Ambient Air Quality Standards.

### Statistical methods

#### Base model

The base model used to analyze each city-specific time series was a Poisson generalized linear model using a framework equivalent to the conditional logistic case-crossover model [18]. This is the same model used by Gass et al. [12]. Time trends were controlled by matching on weekday, month and year, and meteorology was controlled with cubic terms for the three-day moving average of: maximum temperature, maximum temperature interacted with season, and dew point. A spline for day-of-year with two knots was included to provide additional control for seasonal trends. This base model was used to generate the city-specific C&RT trees, estimate the joint effects for the mixtures identified by C&RT, and estimate the joint effects from the comparison multipollutant model.

#### Classification and regression trees: an overview

**Fig. 1:**
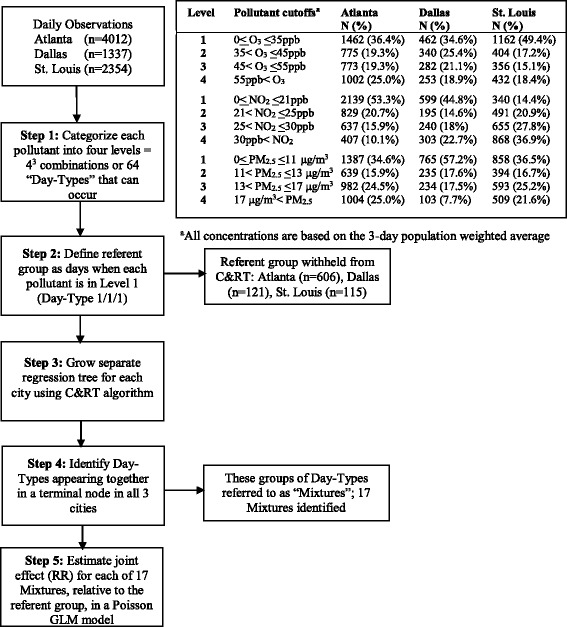
Flow diagram outlining three-city C&RT approach. The table in the upper right contains the concentration cutoffs used to categorize each pollutant into four levels and the frequency at which each level occurred by city

C&RT is a non-parametric regression approach that represents a supervised form of hierarchical clustering in which the observations are sequentially split into dichotomous groups, such that each resulting group contains increasingly similar responses for the outcome [19, 20]. Every tree starts with a “root node” that contains the observations from which the tree will be grown. The observations are then partitioned into two “child nodes” based on the value of an independent predictor variable. The resulting child nodes each contain a subset of the original observations. Child nodes may be further partitioned, again based on the value of an independent predictor variable. This process continues until a set of partitioning criteria are no longer met, resulting in terminal nodes. The collection of terminal nodes forms a complete partition of the observations in the root node. Each terminal node can be viewed as a unique mixture, defined by the path of partitions, or splits, leading from the root node to that particular terminal node. Previously we described how C&RT was used in a single-city analysis [12]; next we describe its application in the three-city analysis. Figure 1 summarizes this approach.

#### Three-city C&RT approach

Step 1: Categorize pollutants into levels: The first step in our three-city C&RT comparison was to specify concentration cut-points for O_3_, NO_2_, and PM_2.5_ that were common across all three cities. Each of the three pollutants was divided into four levels, resulting in 4^3^ or 64 possible daily pollution profiles (aka “Day-Types”). Throughout the paper, the nomenclature used to describe these Day-Types is “O_3_-level/ NO_2_-level/ PM_2.5_-level”. For example, Day-Type “2/2/4” refers to days where both O_3_ and NO_2_ concentrations (3-day moving average) are in the 2^nd^ level and the PM_2.5_ concentration (3-day moving average) is in the 4^th^ level (with levels 1 – 4 ranging from lowest to highest concentration).

Step 2: Define a common referent group: To enable estimation of joint effects, we defined a referent group corresponding to days when all three pollutants were in the lowest level (i.e., days designated as Day-Type 1/1/1). It was decided a priori that the referent group should include at least 100 days in each city. To satisfy this requirement, the referent group was defined as: O_3_ ≤ 35 ppb, NO_2_ ≤ 21 ppb, PM_2.5_ ≤ 11ug/m^3^, which corresponds to roughly the 40^th^ percentile of the overall distribution for the 3-day moving average of each pollutant, from the three cities combined. The remaining level cut-points were defined at approximately the 60^th^ and 80^th^ percentiles (Fig. 1). We chose to use absolute cut-points, rather than city-specific relative cut-points, so improve generalizability of the results. This approach is consistent with the setting of uniform air quality standards across cities or regions.

Step 3: Generate city-specific regression trees: For each city, the referent days were withheld while the remaining days formed the root node in the C&RT algorithm. We considered the nine possible ways these days could be partitioned based on the three pollutant concentration levels. This was accomplished by creating three mutually exclusive indicator variables for each pollutant representing the different comparisons: level 1 vs. 2–4, levels 1 and 2 vs. 3 and 4, and levels 1–3 vs. 4. Each of these nine indicators was considered one-at-a-time in the base model that controlled for confounding (described earlier). The selected indicator was the one that resulted in the smallest *P-*value for the null hypothesis that the beta for that indicator was 0. The days were then partitioned according to pollution levels defined by that indicator. For example, if the indicator representing NO_2_ level 1–3 vs. 4 had the lowest *P*-value, the data were partitioned into two groups: one consisting of all days with NO_2_ concentration in levels 1–3 and the other with days with NO_2_ concentration in level 4. Partitioning according to levels of a single pollutant continued until one of three stopping criteria were met: there were no remaining ways to partition the days, the remaining splits were not significant at a pre-specified level of alpha (α = 0.15), or the minimum number of days for each node (*n* = 60) was not met. In this case partitioning stopped and the node became a terminal node. Note that the confounding variables were included in the model but not used in the splitting.

Step 4: Identify pollution mixtures of interest: We grew three separate trees using the same algorithm and splitting indicator definitions for Atlanta, Dallas, and St. Louis. For each city, the C&RT algorithm partitioned the 63 Day-Types (all but the withheld referent group) into terminal nodes according to their association with the outcome. Rather than comparing the shapes of the trees or the ordering of the splits we focused on the mixtures of air pollutants encompassed in the terminal nodes that were common across cities. For example, suppose Day-Types 2/3/4 and 3/3/4 appear together in a terminal node in all three cities, then we say this pair of Day-Types constitutes a “mixture” in which we are potentially interested. This approach was used to identify all mixtures encompassed in the terminal nodes across the three city trees.

Step 5: Estimate joint effects of mixtures: We then estimated the joint effects of these C&RT-generated mixtures using the base model with indicators for each mixture. Rate ratios for each mixture were estimated using the previously withheld referent group (Day-Type 1/1/1) as the comparison in a three-city model with city-specific effects for all covariates (excluding the mixture indicator variables). Day-Types that did not fall into one of the 17 mixtures were represented with a single indicator variable in the model; rate ratios for these Day-Types are not presented in the Results.

#### Comparison model: multipollutant regression with pollutants modeled continuously

Finally, we compared our findings from the C&RT-identified mixtures to those obtained from a multipollutant regression model with pollutants modeled continuously. This comparison model consisted of the base model described above with the inclusion of a linear term (β*pollutant) for each of the three pollutants, modeled using the continuous 3-day moving average concentrations. A three-city model was run that included city indicator variables and product terms between these indicators and each other model covariate (excluding the pollutant concentrations). To compare the joint effects of this model with those of the C&RT-identified mixtures, we specified pollutant concentrations that were most commensurable to the pollutant concentrations of each mixture as well as the referent group (Day-Type 1/1/1). Specifically, we used the comparison model to estimate the joint effects for a change in concentration from the mean of the referent group to the mean of each C&RT-identified mixture, for each pollutant. This was accomplished by multiplying the coefficient for the linear effect of each pollutant by the difference in the pollutant-specific mixture mean and referent group mean. These products were summed and exponentiated to get the rate ratio for the joint effect. Standard errors for the joint effects were calculated using the variance-covariance matrix. As a sensitivity analysis, we also calculated the joint effects using the contrast represented by the change from the median concentration in the referent group to the median concentration in each C&RT-identified mixture. Further sensitivity analyses were conducted considering alternative comparison models, including a model with all first- and second-order multiplicative terms for the interactions between the pollutants as well as a model with both linear and quadratic terms for each pollutant. As before, joint effects were calculated for the differences in concentration between the mixture mean and the referent group mean. Analyses were performed using SAS® v9.3 (Statistical Analysis System; North Carolina).

## Results

After excluding days with missing air pollution levels, 4012 observations remained for analysis from Atlanta, 2354 days from St. Louis and 1337 days from Dallas. The referent group, identified as days where all pollutants were in the lowest level, contained 606 days (15 %) for Atlanta, 115 days (5 %) for St. Louis, and 121 days (9 %) for Dallas (Fig.1). A more extensive description of the referent group is provided in Table 1, including the monthly distribution, percent of days with precipitation, and average wind speed. Table 2 contains the frequency of each of the 64 Day-Types (the joint distribution of the three pollutants parameterized as ordinal variables) in each city and the correlations between each of the pollutants, by city, are shown in Table 3. All Day-Types occurred at least once in Atlanta; in St. Louis there was one Day-Type that never occurred, and in Dallas there were five Day-Types that never occurred.

**Table 1:** Distribution of referent and non-referent^a^ days by city

	Atlanta				Dallas				St. Louis			
	Referent (*n* = 606)		Non-referent (*n* = 3406)		Referent (*n* = 121)		Non-referent (*n* = 1261)		Referent (*n* = 115)		Non-referent (*n* = 2239)	
Month	N	%	N	%	N	%	N	%	N	%	N	%
January	106	17.49	229	6.72	15	12.40	107	8.80	25	21.74	190	8.49
February	41	6.77	270	7.93	6	4.96	107	8.80	5	4.35	192	8.58
March	26	4.29	315	9.25	14	11.57	110	9.05	1	0.87	203	9.07
April	12	1.98	318	9.34	3	2.48	117	9.62	5	4.35	205	9.16
May	24	3.96	317	9.31	10	8.26	114	9.38	4	3.48	213	9.51
June	15	2.48	315	9.25	13	10.74	107	8.8	0	--	207	9.25
July	9	1.49	332	9.75	8	6.61	116	9.54	0	--	186	8.31
August	18	2.97	323	9.48	2	1.65	122	10.03	4	3.48	182	8.13
September	54	8.91	276	8.10	11	9.09	79	6.50	9	7.83	171	7.64
October	79	13.04	262	7.69	4	3.31	89	7.32	25	21.74	161	7.19
November	96	15.84	234	6.87	11	9.09	79	6.5	21	18.26	159	7.10
December	126	20.79	215	6.31	24	19.83	69	5.67	16	13.91	170	7.59
Precipitation	N	%	N	%	N	%	N	%	N	%	N	%
Days with precipitation	246	40.59	968	28.45	49	40.5	213	17.52	39	33.91	663	29.61
Wind	Mean	SD	Mean	SD	Mean	SD	Mean	SD	Mean	SD	Mean	SD
Wind speed	9.36	3.54	7.62	3.16	12.49	4.62	10.68	4.31	10.03	3.36	8.71	3.21

*SD* standard deviation

^a^Non-referent days were used to generate the city-specific trees

**Table 2:** Frequency at which each Day-Type (*n* = 64) occurred by city, as well as the terminal node designation from the city-specific trees

Day- Type^a^	Atlanta			Dallas			St. Louis		
	N	%	Terminal Node^b^	N	%	Terminal Node^c^	N	%	Terminal Node^d^
1/1/1	606	(15.1 %)	Referent	121	(9.1 %)	Referent	115	(4.9 %)	Referent
1/1/2	137	(3.4 %)	1A	29	(2.2 %)	1D	30	(1.3 %)	6S
1/1/3	98	(2.4 %)	1A	30	(2.2 %)	1D	25	(1.1 %)	6S
1/1/4	24	(0.6 %)	1A	6	(0.4 %)	1D	11	(0.5 %)	1S
1/2/1	137	(3.4 %)	5A	46	(3.4 %)	1D	152	(6.5 %)	3S
1/2/2	56	(1.4 %)	5A	9	(0.7 %)	1D	46	(2 %)	7S
1/2/3	70	(1.7 %)	5A	4	(0.3 %)	1D	63	(2.7 %)	7S
1/2/4	21	(0.5 %)	5A	1	(0.1 %)	1D	26	(1.1 %)	1S
1/3/1	61	(1.5 %)	5A	70	(5.2 %)	1D	171	(7.3 %)	2S
1/3/2	46	(1.1 %)	5A	9	(0.7 %)	1D	47	(2 %)	2S
1/3/3	70	(1.7 %)	5A	5	(0.4 %)	1D	86	(3.7 %)	2S
1/3/4	33	(0.8 %)	5A	0	(0 %)	1D	43	(1.8 %)	2S
1/4/1	11	(0.3 %)	4A	114	(8.5 %)	1D	84	(3.6 %)	2S
1/4/2	23	(0.6 %)	4A	18	(1.3 %)	1D	63	(2.7 %)	2S
1/4/3	41	(1 %)	4A	0	(0 %)	1D	99	(4.2 %)	2S
1/4/4	28	(0.7 %)	4A	0	(0 %)	1D	101	(4.3 %)	2S
2/1/1	221	(5.5 %)	1A	116	(8.7 %)	1D	30	(1.3 %)	3S
2/1/2	92	(2.3 %)	1A	34	(2.5 %)	1D	13	(0.6 %)	6S
2/1/3	104	(2.6 %)	1A	41	(3.1 %)	1D	19	(0.8 %)	6S
2/1/4	25	(0.6 %)	1A	19	(1.4 %)	1D	14	(0.6 %)	1S
2/2/1	80	(2 %)	6A	16	(1.2 %)	1D	47	(2 %)	3S
2/2/2	32	(0.8 %)	6A	10	(0.7 %)	1D	12	(0.5 %)	7S
2/2/3	31	(0.8 %)	6A	5	(0.4 %)	1D	17	(0.7 %)	7S
2/2/4	10	(0.2 %)	6A	1	(0.1 %)	1D	4	(0.2 %)	1S
2/3/1	42	(1 %)	7A	28	(2.1 %)	1D	69	(2.9 %)	2S
2/3/2	21	(0.5 %)	7A	7	(0.5 %)	1D	18	(0.8 %)	2S
2/3/3	31	(0.8 %)	7A	2	(0.1 %)	1D	19	(0.8 %)	2S
2/3/4	16	(0.4 %)	7A	0	(0 %)	1D	3	(0.1 %)	2S
2/4/1	7	(0.2 %)	4A	54	(4 %)	1D	58	(2.5 %)	2S
2/4/2	11	(0.3 %)	4A	6	(0.4 %)	1D	31	(1.3 %)	2S
2/4/3	26	(0.6 %)	4A	0	(0 %)	1D	33	(1.4 %)	2S
2/4/4	26	(0.6 %)	4A	1	(0.1 %)	1D	17	(0.7 %)	2S
3/1/1	123	(3.1 %)	2A	59	(4.4 %)	5D	11	(0.5 %)	3S
3/1/2	88	(2.2 %)	2A	34	(2.5 %)	2D	10	(0.4 %)	6S
3/1/3	154	(3.8 %)	2A	30	(2.2 %)	2D	17	(0.7 %)	6S
3/1/4	80	(2 %)	2A	12	(0.9 %)	2D	12	(0.5 %)	1S
3/2/1	33	(0.8 %)	2A	17	(1.3 %)	5D	24	(1 %)	3S
3/2/2	23	(0.6 %)	2A	11	(0.8 %)	2D	12	(0.5 %)	7S
3/2/3	45	(1.1 %)	2A	16	(1.2 %)	2D	24	(1 %)	7S
3/2/4	39	(1 %)	2A	4	(0.3 %)	2D	14	(0.6 %)	1S
3/3/1	21	(0.5 %)	2A	28	(2.1 %)	6D	31	(1.3 %)	2S
3/3/2	26	(0.6 %)	2A	11	(0.8 %)	2D	18	(0.8 %)	2S
3/3/3	37	(0.9 %)	2A	7	(0.5 %)	2D	23	(1 %)	2S
3/3/4	26	(0.6 %)	2A	1	(0.1 %)	2D	14	(0.6 %)	2S
3/4/1	4	(0.1 %)	2A	35	(2.6 %)	6D	44	(1.9 %)	2S
3/4/2	9	(0.2 %)	2A	7	(0.5 %)	2D	43	(1.8 %)	2S
3/4/3	23	(0.6 %)	2A	6	(0.4 %)	2D	45	(1.9 %)	2S
3/4/4	42	(1 %)	2A	4	(0.3 %)	2D	14	(0.6 %)	2S
4/1/1	22	(0.5 %)	3A	15	(1.1 %)	3D	0	(0 %)	3S
4/1/2	38	(0.9 %)	3A	17	(1.3 %)	3D	7	(0.3 %)	6S
4/1/3	111	(2.8 %)	3A	25	(1.9 %)	3D	7	(0.3 %)	6S
4/1/4	216	(5.4 %)	3A	11	(0.8 %)	3D	19	(0.8 %)	1S
4/2/1	11	(0.3 %)	3A	14	(1 %)	4D	8	(0.3 %)	3S
4/2/2	17	(0.4 %)	3A	10	(0.7 %)	4D	5	(0.2 %)	7S
4/2/3	60	(1.5 %)	3A	26	(1.9 %)	4D	10	(0.4 %)	7S
4/2/4	164	(4.1 %)	3A	5	(0.4 %)	4D	27	(1.1 %)	1S
4/3/1	7	(0.2 %)	3A	15	(1.1 %)	4D	6	(0.3 %)	4S
4/3/2	19	(0.5 %)	3A	15	(1.1 %)	4D	11	(0.5 %)	4S
4/3/3	43	(1.1 %)	3A	24	(1.8 %)	4D	38	(1.6 %)	4S
4/3/4	138	(3.4 %)	3A	18	(1.3 %)	4D	58	(2.5 %)	4S
4/4/1	1	(0 %)	3A	17	(1.3 %)	4D	8	(0.3 %)	5S
4/4/2	1	(0 %)	3A	8	(0.6 %)	4D	28	(1.2 %)	5S
4/4/3	38	(0.9 %)	3A	13	(1 %)	4D	68	(2.9 %)	5S
4/4/4	116	(2.9 %)	3A	20	(1.5 %)	4D	132	(5.6 %)	5S

^a^Day-Types are represented by the level of O_3_, NO_2_ and PM_2.5_ respectively

^b^The seven terminal nodes from the Atlanta tree are designated as 1A – 7A

^c^The six terminal nodes from the Dallas tree are designated as 1D – 6D

^d^The seven terminal nodes from the St. Louis tree are designated as 1S – 7S

**Table 3:** Spearman correlations between O_3_, NO_2_, and PM_2.5_ within each city

City	Pollutant	O_3_	NO_2_	PM_2.5_
	O_3_	1.00	0.19^a^	0.57^a^
Atlanta	NO_2_	0.19^a^	1.00	0.39^a^
	PM_2.5_	0.57^a^	0.39^a^	1.00
	O_3_	1.00	0.05^b^	0.42^a^
Dallas	NO_2_	0.05^b^	1.00	−0.15^a^
	PM_2.5_	0.42^a^	−0.15^a^	1.00
	O_3_	1.00	0.22^a^	0.29^a^
St. Louis	NO_2_	0.22^a^	1.00	0.25^a^
	PM_2.5_	0.29^a^	0.25^a^	1.00

^a^p < 0.01

^b^ *p* = 0.06

**Fig. 2:**
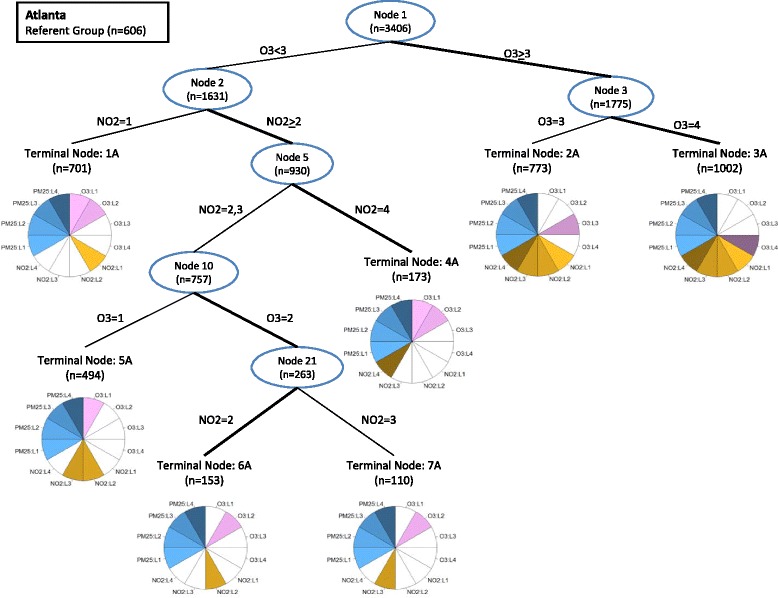
Regression tree illustrating multipollutant joint effects on pediatric asthma emergency department visits in Atlanta (1999 – 2009). Internal nodes are designated with an oval and numbered such that each node, n, produces two child nodes numbers 2n and 2n + 1. The branches of the tree are labeled according to the level of the pollutant used to partition the tree. For each partition, the branch with the more harmful association is bolded. Terminal nodes are numbered 1A-7A (A for Atlanta). The pie graphs at each terminal node are a graphical representation of the Day-Types that fall into each terminal node. Each pie graph has 12 wedges, four representing each level (L1-L4) of O_3_ (shades of purple), four representing each level of NO_2_ (shades of gold), and four representing each level of PM_2.5_ (shades of blue). Pie wedges are colored if a pollutant level is classified into that terminal node and left white if the pollutant level is absent from the terminal node. Day-Types present in the terminal node can be identified by finding every combination of one O_3_ wedge (purple), one NO_2_ wedge (gold) and one PM_2.5_ wedge (blue). For example terminal node 7A contains 4 Day-Types: O_3_ level 2, NO_2_ level 3 and PM_2.5_ levels 1–4 (2/3/1, 2/3/2, 2/3/3, 2/3/4)

**Fig. 3:**
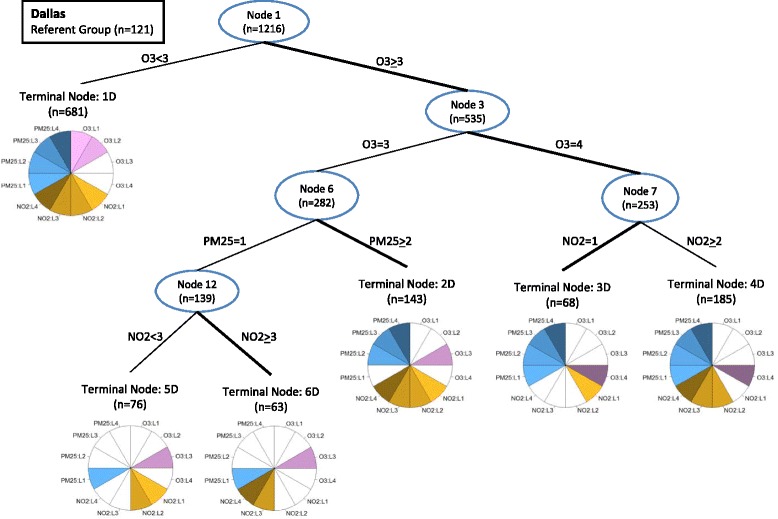
Regression tree illustrating multipollutant joint effects on pediatric asthma emergency department visits in Dallas (2006 –2009). Internal nodes are designated with an oval and numbered such that each node, n, produces two child nodes numbers *2n* and *2n + 1*. The branches of the tree are labeled according to the level of the pollutant used to partition the tree. For each partition, the branch with the more harmful association is bolded. Terminal nodes are numbered 1D-6D (D for Dallas). The pie graphs at each terminal node are a graphical representation of the Day-Types that fall into each terminal node. Each pie graph has 12 wedges, four representing each level (L1-L4) of O_3_ (shades of purple), four representing each level of NO_2_ (shades of gold), and four representing each level of PM_2.5_ (shades of blue). Pie wedges are colored if a pollutant level is classified into that terminal node and left white if the pollutant level is absent from the terminal node. Day-Types present in the terminal node can be identified by finding every combination of one O3 wedge (purple), one NO2 wedge (gold) and one PM_2.5_ wedge (blue). For example terminal node 6D contains 2 Day-Types: O_3_ level 3, NO_2_ levels 3 and 4, and PM_25_ level 1 (3/3/1, 3/4/1)

**Fig. 4:**
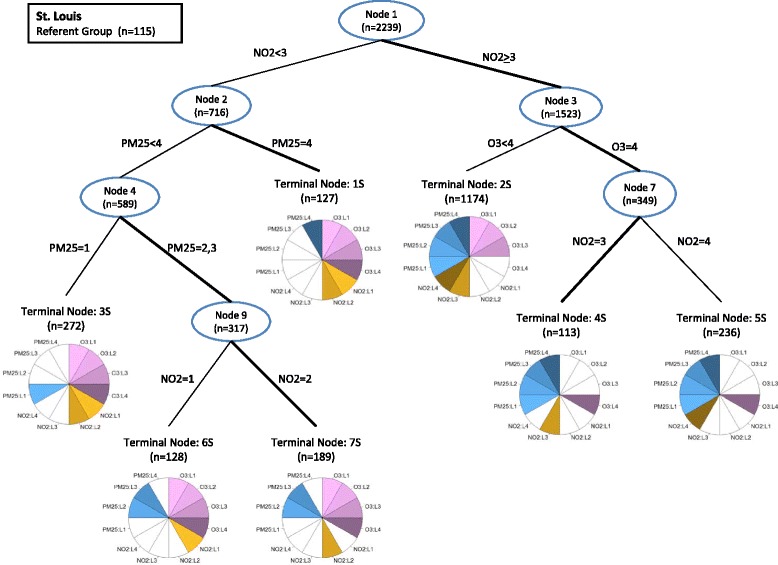
Regression tree illustrating multipollutant joint effects on pediatric asthma emergency department visits in St. Louis (2001–2007). Internal nodes are designated with an oval and numbered such that each node, n, produces two child nodes numbers *2n* and *2n + 1*. The branches of the tree are labeled according to the level of the pollutant used to partition the tree. For each partition, the branch with the more harmful association is bolded. Terminal nodes are numbered 1S-7S (S for St. Louis). The pie graphs at each terminal node are a graphical representation of the Day-Types that fall into each terminal node. Each pie graph has 12 wedges, four representing each level (L1-L4) of O_3_ (shades of purple), four representing each level of NO_2_ (shades of gold), and four representing each level of PM_2.5_ (shades of blue). Pie wedges are colored if a pollutant level is classified into that terminal node and left white if the pollutant level is absent from the terminal node. Day-Types present in the terminal node can be identified by finding every combination of one O_3_ wedge (purple), one NO_2_ wedge (gold) and one PM_2.5_ wedge (blue)

The C&RT algorithm was run separately for each city, generating regression trees with seven, six, and seven terminal nodes in Atlanta, Dallas, and St. Louis, respectively (Figs. 2, 3, 4). Comparing terminal nodes across the three cities, there were 17 pollution mixtures of two or more Day-Types that occurred together in the same terminal node in all three cities (Table 4). The numeric labeling of the mixtures was arbitrary; we ordered the mixtures according to the level of O_3_, followed by NO_2_. Of the 7709 days from the three cities combined, 842 were in the referent group (Day-Type 1/1/1) and 5446 were in one of the 17 mixtures. Table 4 contains the number of days in each mixture by city, as well as the mean concentrations of O_3_, NO_2_, and PM_2.5_. There were 10 Day-Types, corresponding to 1421 days from Atlanta, St. Louis and Dallas combined, that did not appear in any of the 17 mixtures, meaning these Day-Types did not appear together in terminal nodes with other Day-Types consistently across the cities.

**Table 4:** Mixtures described by Day-Type, number of days and mean concentration

Label^a^	Day-Type^b^ O_3_/NO_2_/PM_2.5_	All	Atlanta	Dallas	St. Louis	O_3_^c^	NO_2_^c^	PM_2.5_^c^
		N	N	N	N	Mean(SD)	Mean(SD)	Mean(SD)
Referent	1/1/1	842	606	121	115	25.9 (6.3)	15.4 (3.8)	8.2 (1.7)
Mixture 1	1/1/2, 1/1/3, 2/1/2, 2/1/3	652	431	134	87	32.3 (9.4)	16.4 (3.4)	13.2 (1.6)
Mixture 2	1/1/4, 2/1/4	99	49	25	25	33.1 (10.2)	16.5 (3.5)	19.8 (3)
Mixture 3	1/2/2, 1/2/3	248	126	13	109	23 (7.2)	22.9 (1.2)	13.3 (1.5)
Mixture 4	1/3/1, 1/3/2, 1/3/3, 1/3/4	642	210	85	347	24.8 (7)	27.5 (1.4)	12 (3.9)
Mixture 5	1/4/1, 1/4/2, 1/4/3, 1/4/4, 2/4/1, 2/4/2, 2/4/3, 2/4/4	855	173	196	486	30 (8.9)	34.7 (4.1)	13.3 (5.2)
Mixture 6	2/2/2, 2/2/3	107	63	15	29	40.3 (2.8)	23 (1.1)	13.1 (1.6)
Mixture 7	2/3/1, 2/3/2, 2/3/3, 2/3/4	257	110	38	109	40.1 (2.8)	27.3 (1.4)	11.3 (3.5)
Mixture 8	3/1/1, 3/2/1	267	156	76	35	49.3 (2.8)	18 (4.2)	9.2 (1.4)
Mixture 9	3/1/2, 3/1/3	333	242	64	27	50 (3)	16.3 (3)	13.7 (1.6)
Mixture 10	3/1/4, 3/2/4	161	119	16	26	50.9 (2.8)	18.7 (3.9)	19.9 (2.8)
Mixture 11	3/2/2, 3/2/3	131	68	27	36	49.4 (2.8)	22.9 (1.2)	13.7 (1.6)
Mixture 12	3/3/1, 3/4/1	163	25	63	75	49.7 (2.7)	31 (4.3)	9.1 (1.4)
Mixture 13	3/3/2, 3/3/3, 3/3/4, 3/4/2, 3/4/3, 3/4/4	356	163	36	157	50 (2.9)	31.7 (5.4)	15.6 (4.2)
Mixture 14	4/1/2, 4/1/3,	205	149	42	14	61.4 (5.6)	17.3 (2.8)	14.3 (1.7)
Mixture 15	4/2/2, 4/2/3	128	77	36	15	63.1 (6.1)	23.1 (1.1)	14.3 (1.7)
Mixture 16	4/3/1, 4/3/2, 4/3/3, 4/3/4	392	207	72	113	67.2 (10.7)	27.4 (1.4)	19.1 (6.6)
Mixture 17	4/4/1, 4/4/2, 4/4/3, 4/4/4	450	156	58	236	68.5 (11.2)	35.7 (4.9)	19.7 (6.3)

*SD* standard deviation

^a^Mixture labels are arbitrary and ordered according to level of O_3_, followed by NO_2_

^b^Day-Types are represented by the level of O_3_, NO_2_ and PM_2.5_ respectively

^c^Mean concentrations and standard deviations are presented for the 3-day population weighted average of O_3_ (ppb), NO_2_ (ppb) and PM_2.5_ (μg/m^3^)

The rate ratios (RR) for the 17 mixtures, modeled as indicator variables in the base case-crossover model with all three cities combined, are shown in Table 5 (2^nd^ column). Nearly every RR, with the exception of the RRs for mixtures 1 and 9, was suggestive of a harmful association with pediatric asthma. Mixtures 10 and 13 had the largest effect sizes (RR: 1.07, 95 % CI: 1.03, 1.12; and RR: 1.06, 95 % CI: 1.02, 1.09).

**Table 5:** Rate ratios for the multipollutant joint effects of the C&RT-identified mixtures and comparison model

	C&RT-identified mixtures (modeled with indicators)		Mixture mean - referent mean			Comparison model with continuous terms (linear terms only)	
Label	RR	95 % CI	O_3_	NO_2_	PM_2.5_	RR^a^	95 % CI
Mixture 1	0.99	(0.97, 1.02)	6.35	1.01	5.04	1.01	(1.00, 1.02)
Mixture 2	1.04	(0.99, 1.09)	7.20	1.18	11.64	1.01	(1.00, 1.03)
Mixture 3	1.01	(0.98, 1.04)	−2.89	7.56	5.18	1.01	(1.00, 1.02)
Mixture 4	1.03	(1.01, 1.06)	−1.05	12.11	3.83	1.02	(1.00, 1.03)
Mixture 5	1.04	(1.01, 1.07)	4.06	19.34	5.14	1.04	(1.02, 1.06)
Mixture 6	1.03	(0.99, 1.08)	14.37	7.63	4.97	1.03	(1.02, 1.05)
Mixture 7	1.02	(0.99, 1.06)	14.24	11.91	3.12	1.04	(1.02, 1.06)
Mixture 8	1.04	(1.01, 1.08)	23.43	2.68	0.98	1.04	(1.02, 1.06)
Mixture 9	1.00	(0.97, 1.03)	24.15	0.94	5.56	1.04	(1.02, 1.06)
Mixture 10	1.07	(1.03, 1.12)	24.96	3.35	11.74	1.04	(1.02, 1.07)
Mixture 11	1.04	(0.99, 1.08)	23.48	7.53	5.52	1.05	(1.03, 1.07)
Mixture 12	1.04	(0.99, 1.08)	23.84	15.68	0.92	1.06	(1.04, 1.09)
Mixture 13	1.06	(1.02, 1.09)	24.15	16.29	7.46	1.06	(1.04, 1.09)
Mixture 14	1.01	(0.97, 1.05)	35.53	1.98	6.09	1.06	(1.03, 1.09)
Mixture 15	1.03	(0.98, 1.08)	37.21	7.71	6.16	1.07	(1.04, 1.10)
Mixture 16	1.05	(1.01, 1.09)	41.33	12.04	10.94	1.08	(1.05, 1.12)
Mixture 17	1.05	(1.01, 1.09)	42.56	20.37	11.55	1.10	(1.06, 1.14)
Wald Test^b^	*χ*_*df* = 18_^2^ ^c^ = 36.6, *p* = 0.006					*χ*_*df* = 3_^2^ =27.06, *p* < 0.001	

*C&RT* classification and regression tree, *CI* confidence interval, *DF* degrees of freedom, *RR* rate ratio

^**a**^Rate ratios calculated for the effect of an increase equal to the mixture mean minus the referent mean for all pollutants

^b^Simultaneous Wald test for all exposure parameters in the model

^c^There are 18, as opposed to 17, degrees of freedom to account for the Day-Types not included in any mixture

The RR results of the C&RT-identified mixtures are presented alongside the RRs for the joint effects calculated from the comparison model in Table 5. The RRs shown for the comparison model are for a concentration change from the referent mean to the mixture mean for each of the three pollutants. A Wald test of the statistical significance for the exposure terms, considered simultaneously, was significant for the C&RT model as well as the comparison model (*p* = 0.006 and *p* < 0.001, respectively).

The RRs from the comparison model have relatively good agreement with the C&RT-identified results for the mixtures with lower mean concentrations. At higher pollution levels, joint effects calculated from the comparison model suggest increasing risk with concentration, while the C&RT-identified results suggest risk plateaus at the highest concentration mixtures. For example, the RR for mixture 17, which contains the Day-Type when all pollutants are at their highest level (4/4/4), is 1.05 in the C&RT model (95 % CI: 1.01, 1.09) vs. an RR of 1.10 in the comparison model (95 % CI: 1.06, 1.14). To highlight this further, the 95 % confidence levels for the joint effects of mixtures 1, 9, 14, 15 and 17 calculated using the comparison model do not contain the point estimate from the corresponding joint effects from the C&RT approach, whereas for the other mixtures there was some overlap.

The sensitivity analysis using the mixture medians (as opposed to mean) to calculate the joint effects for the comparison model yielded similar estimates (Table 6). Joint effects from the two alternative comparison models (the multipollutant model with interaction terms and multipollutant model with quadratic effects) using the mixture means are included in Table 6. For the multipollutant model with interaction terms, the confidence intervals were large, while for the quadratic model, a test of significance for the exposure terms beyond the linear effects was non-significant (*p* = 0.63) and suggests that the multipollutant effect does not have a quadratic form. In both sensitivity analysis models, the point estimates of the joint effects for each mixture were nearly identical to the comparison model with only linear effects.

**Table 6:** Sensitivity analysis results for linear term models using Mixture median and Mixture mean with interactions and quadratic terms

			Comparison models with continuous terms					
	C&RT-identified mixtures (modeled with indicators)		Linear terms (mixture median)		Linear terms + all two- and three-way interaction terms (mixture mean)		Linear terms + quadratic effects (mixture mean)	
Label	RR	95 % CI	RR^a^	95 % CI	RR^b^	95 % CI	RR^b^	95 % CI
Mixture 1	0.99	(0.97, 1.02)	1.01	(1.00, 1.02)	1.02	(0.96, 1.08)	1.01	(1.00, 1.03)
Mixture 2	1.04	(0.99, 1.09)	1.02	(1.00, 1.03)	1.03	(0.97, 1.11)	1.02	(1.00, 1.04)
Mixture 3	1.01	(0.98, 1.04)	1.01	(1.00, 1.02)	1.01	(0.97, 1.06)	1.01	(1.00, 1.03)
Mixture 4	1.03	(1.01, 1.06)	1.02	(1.00, 1.03)	1.02	(0.97, 1.07)	1.02	(1.01, 1.04)
Mixture 5	1.04	(1.01, 1.07)	1.03	(1.01, 1.05)	1.04	(0.98, 1.10)	1.04	(1.02, 1.06)
Mixture 6	1.03	(0.99, 1.08)	1.03	(1.02, 1.04)	1.04	(0.97, 1.12)	1.04	(1.02, 1.06)
Mixture 7	1.02	(0.99, 1.06)	1.04	(1.02, 1.05)	1.05	(0.98, 1.13)	1.05	(1.02, 1.07)
Mixture 8	1.04	(1.01, 1.08)	1.04	(1.02, 1.06)	1.05	(0.96, 1.14)	1.04	(1.02, 1.07)
Mixture 9	1.00	(0.97, 1.03)	1.04	(1.02, 1.06)	1.05	(0.96, 1.15)	1.04	(1.02, 1.07)
Mixture 10	1.07	(1.03, 1.12)	1.04	(1.02, 1.07)	1.06	(0.97, 1.15)	1.05	(1.02, 1.08)
Mixture 11	1.04	(0.99, 1.08)	1.05	(1.03, 1.07)	1.06	(0.97, 1.15)	1.05	(1.03, 1.08)
Mixture 12	1.04	(0.99, 1.08)	1.06	(1.03, 1.08)	1.08	(0.99, 1.18)	1.06	(1.03, 1.09)
Mixture 13	1.06	(1.02, 1.09)	1.06	(1.04, 1.08)	1.08	(0.99, 1.17)	1.07	(1.04, 1.10)
Mixture 14	1.01	(0.97, 1.05)	1.06	(1.02, 1.08)	1.07	(0.96, 1.19)	1.06	(1.03, 1.10)
Mixture 15	1.03	(0.98, 1.08)	1.07	(1.03, 1.10)	1.08	(0.97, 1.21)	1.08	(1.04, 1.11)
Mixture 16	1.05	(1.01, 1.09)	1.08	(1.04, 1.11)	1.09	(0.98, 1.22)	1.09	(1.05, 1.13)
Mixture 17	1.05	(1.01, 1.09)	1.09	(1.05, 1.13)	1.10	(0.98, 1.24)	1.10	(1.06, 1.15)
Wald Test^c^	*χ*_*df* = 18_^2^ =36.6, p = 0.006		*χ*_*df* = 3_^2^ =27.06, p < 0.001		*χ*_*df* = 7_^2^ =40.69, p < 0.001		*χ*_*df* = 6_^2^ =28.77, p < 0.001	
Wald Test^d^	*NA*		*NA*		*χ*_*df* = 4_^2^ =13.55, p = 0.009		*χ*_*df* = 3_^2^ =1.72, p = 0.633	

*C&RT* classification and regression tree, *CI* confidence interval, *DF* degrees of freedom, *RR* rate ratio

^**a**^Rate ratios for the comparison model are calculated for the effect of an increase equal to the mixture median minus the referent median for all three pollutants

^**b**^Rate ratios for the comparison model are calculated for the effect of an increase equal to the mixture mean minus the referent mean for all three pollutants

^c^Simultaneous Wald test for all exposure parameters in the model

^d^Simultaneous Wald test for additional exposure parameters beyond the linear effects

## Discussion

In this paper we used classification and regression trees, a non-parametric recursive partitioning approach, to identify multipollutant joint effects associated with pediatric asthma in Atlanta, Dallas and St. Louis. As evidenced by our sensitivity analyses, it is difficult to identify complex interactions of two, three or four pollutants using conventional regression models due to power limitations [21]. A known advantage of C&RT is that it can be used to detect complex and multiple interactions between covariates [8, 9]. We have previously shown that with few modifications, C&RT can be used to detect interactions between pollutant concentrations while simultaneously controlling for temporal and meteorological confounding [12].

A key finding of this analysis is that no single C&RT-identified mixture emerged as being substantially more harmful than the rest. All mixtures, with the exception of mixtures 1 and 9, had a harmful association relative to days when all pollutants were in the lowest level. There were seven mixtures with statistically significant associations, all of which were similar in magnitude. There was no apparent pollutant-specific pattern to the RRs of the C&RT-identified mixtures, suggesting that no single pollutant was driving the associations. Instead the results of the C&RT approach suggest that higher levels of any of the three pollutants (O_3_, NO_2_, and PM_2.5_) are more harmful and that the rates appear to plateau at higher concentrations. For example mixture 17, which contains the highest mean concentrations of O_3_ and NO_2_ and the second highest PM_2.5_ concentration, has an RR of 1.05, while mixture 13, which is characterized by more moderate concentrations of the three pollutants, has an RR of 1.06. While this lack of a synergistic --or even multiplicative-- response is surprising, it is not unprecedented. In a review of the literature, Mauderly and Samet found that 22 out of 36 laboratory studies failed to demonstrate a synergistic response [22]. A hypothesis that could explain the C&RT finding that risk plateaus on the highest pollution days is if asthmatic children change their behavior and limit exposure. A cross-sectional study by Wen et al. lends some support to this theory; the study found that asthmatic adults had a greater odds of modifying their outdoor activity compared with non-asthmatics on days with media alerts due to a high air quality index [23].

This C&RT approach for identifying and evaluating multipollutant joint effects yielded different results than the multipollutant comparison model with continuous terms for the three pollutants. This latter multipollutant regression model was selected for comparison with the C&RT approach because it is an extension of the traditional single-pollutant models and likely represents a characterization of joint effects for which researchers are most familiar, although only a few studies have actually used such models to estimate a combined multipollutant joint effect [16, 24–26]. It should be emphasized that the comparison model was not expected to generate equivalent results as the C&RT model, but rather to serve as a comparison for the C&RT approach.

Examination of the differences between the C&RT and comparison model results suggests that the two approaches for modeling multipollutant exposures lead to different conclusions regarding the roles of individual pollutants. In the comparison model, joint effects are driven by O_3_ concentration. This is demonstrated by the increasing RRs from top to bottom in Table 5, an artifact of assigning arbitrary labels to the mixtures based on increasing O_3_ concentration. Conversely, one does not see the same pattern of increasing RRs in the C&RT-identified mixtures, again suggesting that no single pollutant is driving these results. With respect to PM_2.5_, results from the comparison model with continuous terms suggest that PM_2.5_ has a null association (overall RR 1.00 for an IQR increase in PM_2.5,_ holding the other two pollutants constant), while the C&RT approach suggests that PM_2.5_ drives some of the mixture associations. This is perhaps best highlighted by comparing the RRs for mixtures 1 and 2, which have very similar levels of O_3_ and NO_2_ but differ in the concentration of PM_2.5_. Using the C&RT approach the RR for mixture 1, which has the lower PM_2.5_ mean concentration, is 0.99 (95 % CI: 0.97, 1.02) while the RR for mixture 2 is 1.04 (95 % CI: 0.99, 1.09) (Table 5). In contrast, the RRs for mixtures 1 and 2 using the multipollutant comparison model are both 1.01 (with 95 % CIs of 1.00, 1.02 and 1.00, 1.03, respectively).

Although the joint effects of the C&RT and comparison models differ (i.e., in the C&RT model pollution is modeled categorically whereas in the comparison model pollution is modeled continuously), the differences implied by the results are striking and merit further attention. One possibility is that C&RT identified joint effects are driven by PM_2.5_ constituents. For example, the comparison model treats all specific PM_2.5_ concentrations equally; a high PM_2.5_ day that has high levels of elemental carbon is not distinguished from a high day that has a greater proportion of sulfates. Conversely the C&RT model has the potential to distinguish mixtures of PM_2.5_ constituents through the interactions detected by subsequent partitioning. By partitioning on a pollutant (e.g., NO_2_) that is correlated with certain PM_2.5_ components, C&RT has the ability to differentiate PM_2.5_ mixtures through their correlation with other independent variables in the model. While the multipollutant comparison model with the inclusion of first- and second-order interaction terms could discriminate between some PM_2.5_ mixtures, its discriminatory power would be limited to a linear effect for each of the interaction terms. As such it could not, for example, identify the same complex interactions as seen in the St. Louis C&RT tree through nodes 1-2-4-9 (Fig.4).

By binning days, the C&RT model may be able to account for unmeasured confounding that is non-smooth (i.e., that varies with terminal node classification, not pollution). For example, people may modify their behavior under certain types of days in a way that affects ED visits for asthma. As a result, it is possible that the point estimates for the mixture results are measuring not only the multipollutant effect but also the effects of other factors that are correlated with those Day-Types. While this could be a disadvantage if one intends to use the point estimates to conduct risk assessment, it could be beneficial if the interest is in identifying types of days that are most harmful for a particular health outcome and could lead to a more targeted warning system.

When considering the C&RT approach, if two or more Day-Types appear together in a terminal node, this is either indicative of homogeneity of effect or lack of power to detect any further effect (i.e., drive any further partitioning). It is likely that one or more of the 17 mixtures were formed as a result of insufficient power to further partition the terminal node in a given city. This would result in an “artificial group”, that is a mixture in which the Day-Types do not have a similar association with the outcome.

One downside to presenting the combined mixture RRs in this analysis is that any heterogeneity across the cities will be masked. Some between-city heterogeneity is to be expected due to tangible and intangible city differences, including socio-economic status, air conditioning use, climate acclimation and behavior patterns that are likely to modify the estimated health associations [27]. Nonetheless, it seems likely that there exist some ambient pollution mixtures that are universally harmful, and by combining cities our aim was to overcome some of the power limitations mentioned above.

## Conclusion

C&RT can be used to investigate multipollutant joint effects and may lead to different conclusions than multipollutant models with continuous terms. In particular, the results from this study suggest C&RT and comparison models lead to different joint effects of O_3_, NO_2_ and PM_2.5_ when concentrations are high and that the apparent role of PM_2.5_ differs according to the model used. Furthermore we have shown how C&RT models can be used to identify types of days that are particularly harmful to health, which can help to improve warning systems and lead to more targeted regulation.

Understanding the potential risk air pollution mixtures pose to human health is a complex and challenging undertaking that has only just begun. It is important to emphasize that we do not know which, if either, of the models is more correct. Different assumptions are made when exposure is modeled categorically vs. continuously, making direct comparisons of results difficult. Nevertheless, exploring alternative models can be a useful way to generate new ideas and perhaps gain greater insight into air pollution mixtures.

## Abbreviations

CI: Confidence interval
C&RT: Classification and regression trees
ED: Emergency Department
NO2: Nitrogen dioxide
O3: Ozone
PM2.5: Particulate Matter (<2.5 μg)
RR: Rate ratio
